# Timing of additional neoadjuvant chemotherapy in patients with locally advanced rectal cancer treated with neoadjuvant chemoradiotherapy and total mesorectal excision

**DOI:** 10.1007/s12672-022-00572-4

**Published:** 2022-10-21

**Authors:** Fang He, Mo Chen, Yan-ping Liu, Jiachun Sun, Jian Zheng

**Affiliations:** 1 Department of Radiation Oncology, Guangdong Provincial Key Laboratory of Colorectal and Pelvic Floor Diseases, The Sixth Affiliated Hospital, Sun Yat-Sen University, 26 YuanCun ErHeng Road, Guangzhou, Guangdong 510655 People’s Republic of China; 2 Department of Radiation Oncology, Cancer Center, The First People’s Hospital of Foshan, Foshan, Guangdong People’s Republic of China; 3 Department of Pathology, Henan Key Laboratory of Cancer Epigenetics, The First Affiliated Hospital, and College of Clinical Medicine of Henan University of Science and Technology, No. 24, Jinhua Road, Jianxi District, Luoyang, Henan 471003 People’s Republic of China

**Keywords:** Consolidation chemotherapy, Induction chemotherapy, Paradigm, Survival, Rectal cancer

## Abstract

**Background:**

In locally advanced rectal cancer (LARC), the optimal sequence of neoadjuvant chemotherapy in relation to neoadjuvant chemoradiotherapy and before total mesorectal excision is unknown.

**Methods:**

A total of 426 LARC patients, treated with neoadjuvant chemoradiotherapy followed by total mesorectal excision, between January 2010 and December 2018, were studied retrospectively. Patients were divided into induction and consolidation chemotherapy groups. Overall, disease-free, locoregional relapse-free, and distant metastasis-free survival rates for the 2 groups were compared. Multivariate analysis hazard ratios (HR) with 95% confidence intervals (CI) to identify survival predictors.

**Results:**

Median follow-up was 37 (range, 7–162) months. The 3-year overall, disease-free, locoregional relapse-free, and distant metastasis-free survival rates were 93.8%, 71.6%, 93.5%, and 74.4%, respectively. For those receiving either induction or consolidation chemotherapy, 3-year disease-free survival rates were 82.5% and 67.7%, respectively (*P* = 0.021), distant metastasis-free rates were 85.4% and 70.8%, respectively (*P* = 0.024), and both overall and locoregional relapse-free survival rates did not differ significantly. Absence of neural invasion was an independent predictor of disease-free (HR = 0.49, 95% CI 0.25–0.97, *P* = 0.04) and distant metastasis-free (HR = 0.49, 95% CI 0.25–0.98, *P* = 0.04) survival. Both ypTN stage III (vs.0-II) and consolidation (vs. induction) chemotherapy were independent predictors of disease relapse (HR = 1.95, 95% CI 1.47–2.58, *P* < 0.001; HR = 1.68, 95% CI 1.01–2.79, *P* = 0.046; respectively) and distant metastasis (HR = 2.04, 95% CI 1.51–2.76, *P* < 0.001; HR = 1.75, 95% CI 1.03–2.99, *P* = 0.04; respectively).

**Conclusions:**

LARC patients receiving neoadjuvant chemoradiotherapy and total mesorectal excision had better disease-free and distant metastasis-free survival, with induction rather than consolidation neoadjuvant chemotherapy.

## Introduction

The standard treatment for locally advanced rectal cancer (LARC) has been neoadjuvant chemoradiotherapy (nCRT) with total mesorectal excision (TME) surgery, often followed by adjuvant chemotherapy (ACT) [1]. Although this trimodal approach has led to a significant reduction in local recurrence rates, distant metastases are the main cause of death, occurring in approximately 30% of these patients [2–4]. In addition, treatment with ACT has failed to improve disease-free survival (DFS) and overall survival (OS), and its use is frequently compromised by poor patient tolerance and the need for marked dose reductions [5–7].

Consequently, recent treatment strategies for patients with LARC have focused instead on giving additional cycles of chemotherapy prior to TME [2, 8]. The intensification of preoperative treatment with standard dose polychemotherapy administration before surgery, known as total neoadjuvant treatment (TNT), has shown encouraging results [9]. Moreover, the improvement in radiological field could allow to predict pathological response after neoadjuvant treatment; in particular, the assessment of 18F-FDG PET-CT uptake decrease, could guide the choice of administered therapy [10]. In a meta-analysis involving 28 studies, patients with LARC receiving neoadjuvant chemotherapy (NCT) in addition to nCRT had better DFS and OS rates than those receiving nCRT alone [11].

Although NCT is being used increasingly in clinical practice for patients with LARC, the optimal scheduling of NCT and nCRT before TME remains uncertain. The recent CAO/ARO/AIO-12 trial has suggested that the sequencing of NCT and nCRT does seem to affect patient outcomes, demonstrating that nCRT followed by consolidation NCT resulted in the highest pathologic complete response (pCR) rate and clinical complete response (cCR), also reducing grade 3–4 AEs [12]. However, others have noted that it is unknown whether improved pCR rates actually translate into improved survival [13]. The PRODIGE 23 phase III randomized trial [14], has suggested that induction NCT and a shorter course of ACT demonstrated significantly better 3-year DFS (75.7% vs. 68.5%, *P* = 0.03) and DMFS (78.8% vs. 71.7%, *P* < 0.02) rates, compared to the group who received the longer course of conventional ACT without NCT. Meanwhile, in the RAPIDO trial [15], compared to the group not receiving NCT, the group receiving consolidation NCT showed a significantly lower risk of disease-related treatment failure (30.4% vs. 23.7%, P = 0.02) and distant metastasis (26.6% vs. 19.8%, P = 0.004). Recently, in the STELLAR trial [16], short-term radiotherapy with preoperative chemotherapy followed by surgery showed noninferiority treatment efficacy compared with standard preoperative nCRT group with regard to disease-free survival (64.5% *v* 62.3%; *P* < 0.001 for noninferiority). Additional investigation into the best timing for NCT is still warranted [17].

We were interested in determining the preferable sequence of timing of NCT relative to nCRT in patients with LARC. To explore this issue, we conducted a retrospective study involving patients with LARC who received NCT, nCRT, and TME at our hospital over an 8-year period. We compared the survival rates of patients who were treated with one of two different NCT paradigms, either induction chemotherapy prior to nCRT or nCRT followed by consolidation chemotherapy.

## Materials and methods

The Institutional Review Board of the Sixth Affiliated Hospital at Sun Yat-sen University approved this retrospective study. The study protocol was approved by the Central Ethics Committee of The Sixth Affiliated Hospital, Sun Yat-sen University (Guangzhou, China) (No. 2020ZSLYEC-289).

### Patient selection

**Fig. 1:**
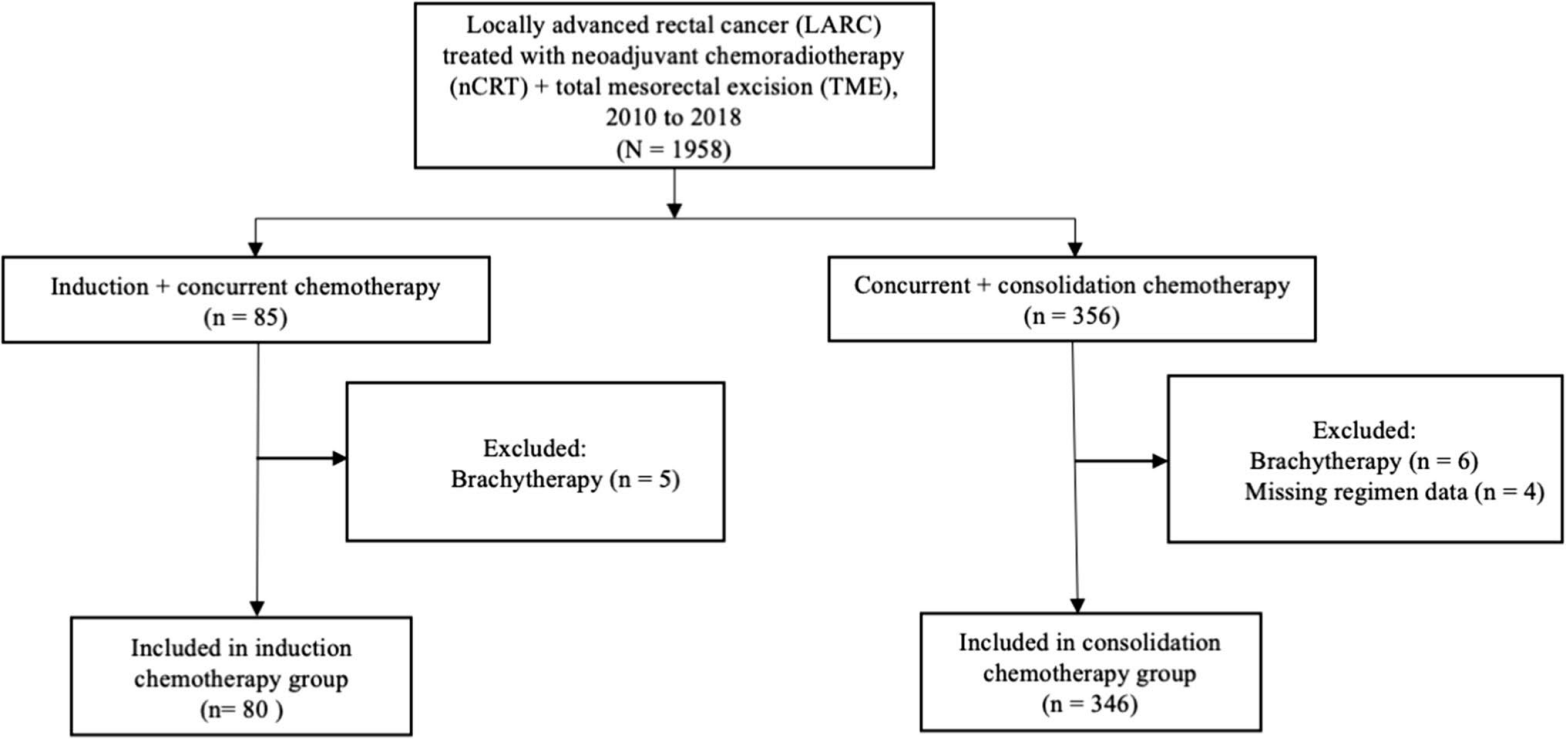
CONSORT flow diagram for study of 426 patients with locally advanced rectal cancer (LARC), who had different neoadjuvant chemotherapy paradigms (induction vs. consolidation) as part of neoadjuvant chemoradiotherapy (nCRT) prior to total mesorectal excision (TME), January 2010 through December 2018

We conducted a retrospective study of consecutive patients with biopsy-proven, locally advanced, non-metastatic rectal cancer who had been treated with nCRT followed by TME at our center between January 2010 and December 2018 (Fig. 1). Of the 1,958 patients who were initially identified, 426 (21.8%) met the following inclusion criteria: (a) had clinical stage II (T3-4N0) or stage III (T1-4N1-2) rectal cancer, (b) had no distant metastasis, and (c) had undergone NCT prior to TME, either induction chemotherapy before nCRT (IC group) or consolidation chemotherapy after nCRT (CC group). Patients were excluded from the study if they had a histologic type of rectal cancer other than adenocarcinoma, had refused surgery or TME with palliative rather than curative intent, had unfinished neoadjuvant radiotherapy course, received brachytherapy, or had missing data about the chemotherapy regimens received.

### Neoadjuvant and adjuvant treatment

All patients in the study received NCT. Patients in the IC group received induction (before radiotherapy) and concurrent (during radiotherapy) chemotherapy; those in the CC group received concurrent (during radiotherapy) and consolidation (after radiotherapy) chemotherapy. A majority but not all of the patients in the study also received ACT, which was given after TME, and was chosen at the discretion of the multi-disciplinary cancer team at our hospital. All patients in this study received a total of at least 8 preoperative chemotherapy cycles. The chemotherapy regimens used consisted of a fluoropyrimidine-based regimen. Chemotherapy typically involved a fluoropyrimidine-based regimen, consisting of one or more of the following: folinic acid with fluorouracil (de Gramont) and oxaliplatin (FOLFOX); capecitabine with oxaliplatin (CAPOX), or capecitabine (Xeloda) alone. Concurrent chemotherapy regimens always included oral capecitabine.

All patients received intensity-modulated radiotherapy (IMRT), and they were treated 5 days a week with 1 fraction daily. The radiotherapy was provided as follows: a total dose of 50 Gy to the planning target volume (PTV) of the gross tumor volume of the primary rectal tumor (GTVp), given in 25 fractions at 2.0 Gy per fraction; 45 Gy to the PTV of the clinical target volume (CTV), given in 25 fractions at 1.8 Gy per fraction. The CTV was defined as the GTV plus areas considered at significant risk of harboring microscopic disease, including the mesorectum (perirectal fascia), perirectal nodes, presacral region, and internal iliac lymph node region. External iliac nodes were included if the primary tumor invaded adjacent organs (cT4) or if the obturator nodes or external iliac nodes were involved.

### Population characteristics

Disease staging was determined at the time of pretreatment evaluation by magnetic resonance imaging (MRI), computed tomography (CT), and/or endorectal ultrasonography (EUS). All patients were staged according to the 7th edition of the American Joint Commission on Cancer (AJCC) staging system [18].

Baseline characteristics evaluated in this study included the following: age, gender, ECOG grade, clinical TNM stage, distance of distal tumor from anal verge, and tumor differentiation. Clinicopathological characteristics included the following: pathologic stage after nCRT and TME (ypTN stage); pathologic complete response (pCR), defined as the absence of viable adenocarcinoma cells in the TME surgical specimen histopathology (ypT0N0); and other post-TME histopathology results (vascular invasion, neural invasion, surgical margin, and circumferential resection margin).

### Follow-up and outcomes

Follow-up duration was defined as the time from the first day of any treatment to either the date of last examination or the date of death. Patients were routinely assessed at 3-month intervals during the first 3 years of care and at 6-month intervals thereafter. Primary outcomes used in this study included the following: OS, measured as the time from the initiation of treatment to death from any cause; DFS, measured as the time from TME to the first disease relapse at any site; locoregional relapse-free survival (LRFS), measured as the time from TME to the first locoregional (within the pelvis) relapse; and distant metastasis-free survival (DMFS), measured as the time from TME to the first distant (outside the pelvis) relapse.

### Statistical methods

The χ2 test was used to compare the distributions of assorted demographic and clinicopathological characteristics for the IC and CC groups. All survival outcome measures were censored on April 27, 2022. Kaplan–Meier survival curves were used to compare patient survival outcomes between two groups. Statistical differences between curves were calculated using the log-rank test. The multivariate Cox proportional hazards model was utilized to assess potentially relevant factors derived from univariate analysis. The hazard ratios (HR) and 95% confidence intervals (CI) were also calculated. All *P* values were two-sided, and a *P*-value less than 0.05 was considered statistically significant. Statistical analyses were performed with SPSS (version 24.0; SPSS, Inc, Chicago, IL).

## Results

### Comparison of NCT paradigms

Of the 426 patients included in this study, 80 (18.8%) were in the IC group and 346 (81.2%) were in the CC group. The median age of all patients was 58.0 (range, 20–87) years (Table 1). There were no statistical differences between the two groups in gender, ECOG grade, clinical N stage, clinical TNM stage, distance of distal tumor from anal verge, or tumor differentiation. A larger proportion of patients in the IC group were young (68.8% vs. 46.5% were 58 years old and younger), whereas a larger proportion of patients in the CC group were older (53.5% vs. 31.3% were 59 years old and older), and these differences were significant (*P* < 0.001). Additionally, 40.0% of patients in the IC group had clinical stage T4 rectal cancer whereas only 15.3% of those in the CC group had clinical stage T4 disease (*P* < 0.001).

**Table 1:** Baseline characteristics of 426 patients with locally advanced rectal cancer, by different neoadjuvant chemotherapy paradigms as part of neoadjuvant chemoradiotherapy prior to total mesorectal excision, January 2010 through December 2018

Characteristics	Neoadjuvant chemotherapy paradigms		*P*
	Induction	Consolidation	
	n (%)	n (%)	
Total patients	80 (100.0)	346 (100.0)	–
Age, *years* (median, 58)			< 0.001
≤ 58	55 (68.8)	161 (46.5)	
> 58	25 (31.3)	185 (53.5)	
Gender			0.53
Male	50 (62.5)	229 (66.2)	
Female	30 (37.5)	117 (33.8)	
ECOG grade^a^			0.11
0	30 (37.5)	161 (46.5)	
1	47 (58.8)	181 (52.3)	
2	3 (3.7)	4 (1.2)	
Clinical T stage			< 0.001
cT2	1 (1.2)	18 (5.2)	
cT3	47 (58.8)	275 (79.5)	
cT4	32 (40.0)	53 (15.3)	
Clinical N stage			0.81
cN0	16 (20.0)	70 (20.2)	
cN1	31 (38.8)	146 (42.2)	
cN2	33 (41.3)	130 (37.6)	
Clinical TNM stage			0.96
II	16 (20.0)	70 (20.2)	
III	64 (80.0)	276 (79.8)	
Distance of distal tumor from anal verge, *cm*			0.19
0 to 5	43 (53.8)	176 (50.9)	
> 5 to ≤ 10	31 (38.8)	158 (45.7)	
> 10	6 (7.5)	12 (3.5)	
Tumor differentiation^b^			0.52
High	21 (26.3)	71 (20.5)	
Moderate	38 (47.5)	182 (52.6)	
Poor	21 (26.3)	93 (26.9)	
Mesorectal fascia			0.14
Negative	8 (10)	63 (18.2)	
Positive	48 (60)	173 (50)	
Missing	24 (40)	110 (31.8)	

^a^Eastern Cooperative Oncology Group (ECOG) performance status

^b^Based on histopathology after total mesorectal excision

The proportion of patients receiving more than 4 NCT cycles was significantly higher in the IC group than in the CC group (36.3% vs. 5.5%, *P* < 0.001) (Table 2). On the other hand, the proportion of patients receiving 5 or more ACT cycles was not significantly different in the IC and CC groups (40.0% vs. 50.0%, *P* = 0.26). Ultimately, the median total number of chemotherapy cycles received by patients in the 2 groups were the same. The median intervals between radiotherapy and TME (56.5 days vs. 56.0 days) were also similar.

**Table 2:** Chemotherapy-related characteristics of 426 patients with locally advanced rectal cancer, by different neoadjuvant chemotherapy paradigms as part of neoadjuvant chemoradiotherapy prior to total mesorectal excision (TME), January 2010 through December 2018

Characteristics	Neoadjuvant chemotherapy paradigms		*P*
	Induction	Consolidation	
Total patients, n (%)	80 (100.0)	346 (100.0%)	–
Neoadjuvant chemotherapy cycles, n (%)			< 0.001
> 1 to ≤ 4	51 (63.7)	327 (94.5)	
> 4 to ≤ 8	29 (36.3)	18 (5.2)	
> 8 to ≤ 12	0 (0.0)	1 (0.3)	
Adjuvant chemotherapy cycles, n (%)			0.26
0	10 (12.5)	40 (11.6)	
1 to 4	38 (47.5)	133 (38.4)	
≥ 5	32 (40.0)	173 (50.0)	
Total chemotherapy cycles			–
Median (range)	8 (3,12)	8 (3,12)	
Interval between radiotherapy and TME, *days*			–
Median (range)	56.5 (28,129)	56.0 (29,176)	

The proportion of patients in the IC and CC groups with pCR were not significantly different (22.5% vs. 18.5%, *P* = 0.41) (Table 3). The rate of downstaging in the two groups was also not significantly different (51.2% vs. 40.5%, *P* = 0.08). Conversely, a higher proportion of patients in the IC group than in the CC group ultimately had stage ypT4 stage disease (10.0% vs. 4.1%, P = 0.041). The proportion of patients with positive surgical margins in both groups were low and did not differ significantly (0.0% in the IC group and 0.6% in the CC group, *P* = 0.50).

**Table 3:** Clinicopathological outcomes of 426 patients with locally advanced rectal cancer, by different neoadjuvant chemotherapy paradigms as part of neoadjuvant chemoradiotherapy prior to total mesorectal excision, January 2010 through December 2018

Outcomes	Neoadjuvant chemotherapy paradigms		*P*
	Induction	Consolidation	
	n (%)	n (%)	
Total patients	80 (100.0)	346 (100.0)	–
Pathologic complete response (pCR)^a^			0.41
yes	18 (22.5)	64 (18.5)	
no	62 (77.5)	282 (81.5)	
ypT stage^b^			0.04
ypT0	19 (23.7)	68 (19.7)	
ypT1	6 (7.5)	23 (6.6)	
ypT2	21 (26.3)	71 (20.5)	
ypT3	26 (32.5)	170 (49.1)	
ypT4	8 (10.0)	14 (4.1)	
ypN stage^b^			0.91
ypN0	60 (75.0)	251 (72.5)	
ypN1	17 (21.3)	81 (23.5)	
ypN2	3 (3.7)	14 (4.0)	
ypTN stage^b^			0.34
ypT0N0	18 (22.5)	64 (18.5)	
I	23 (28.7)	76 (22.0)	
II	20 (25.0)	115 (33.2)	
III	19 (23.8)	91 (26.3)	
Downstaging (to stage ypT0-2N0^b^)			0.08
yes	41 (51.2)	140 (40.5)	
no	39 (48.8)	206 (59.5)	
Vascular invasion^c^			0.55
negative	79 (98.8)	338 (97.7)	
positive	1 (1.2)	8 (2.3)	
Neural invasion^c^			0.20
negative	78 (97.5)	325 (93.9)	
positive	2 (2.5)	21 (6.1)	
Surgical margin^c^			0.50
negative	80 (100.0)	344 (99.4)	
positive	0 (0.0)	2 (0.6)	
Circumferential resection margin^c^, *mm*			0.24
> 1	80 (100.0)	340 (98.3)	
≤ 1	0 (0.0)	6 (1.7)	

^a^Pathological complete response (pCR) equivalent to pathologic stage ypT0N0

^b^The yp stages are pathologic stages after neoadjuvant chemoradiotherapy and total mesorectal excision

^c^Based on histopathology after total mesorectal excision

### Outcomes

**Fig. 2:**
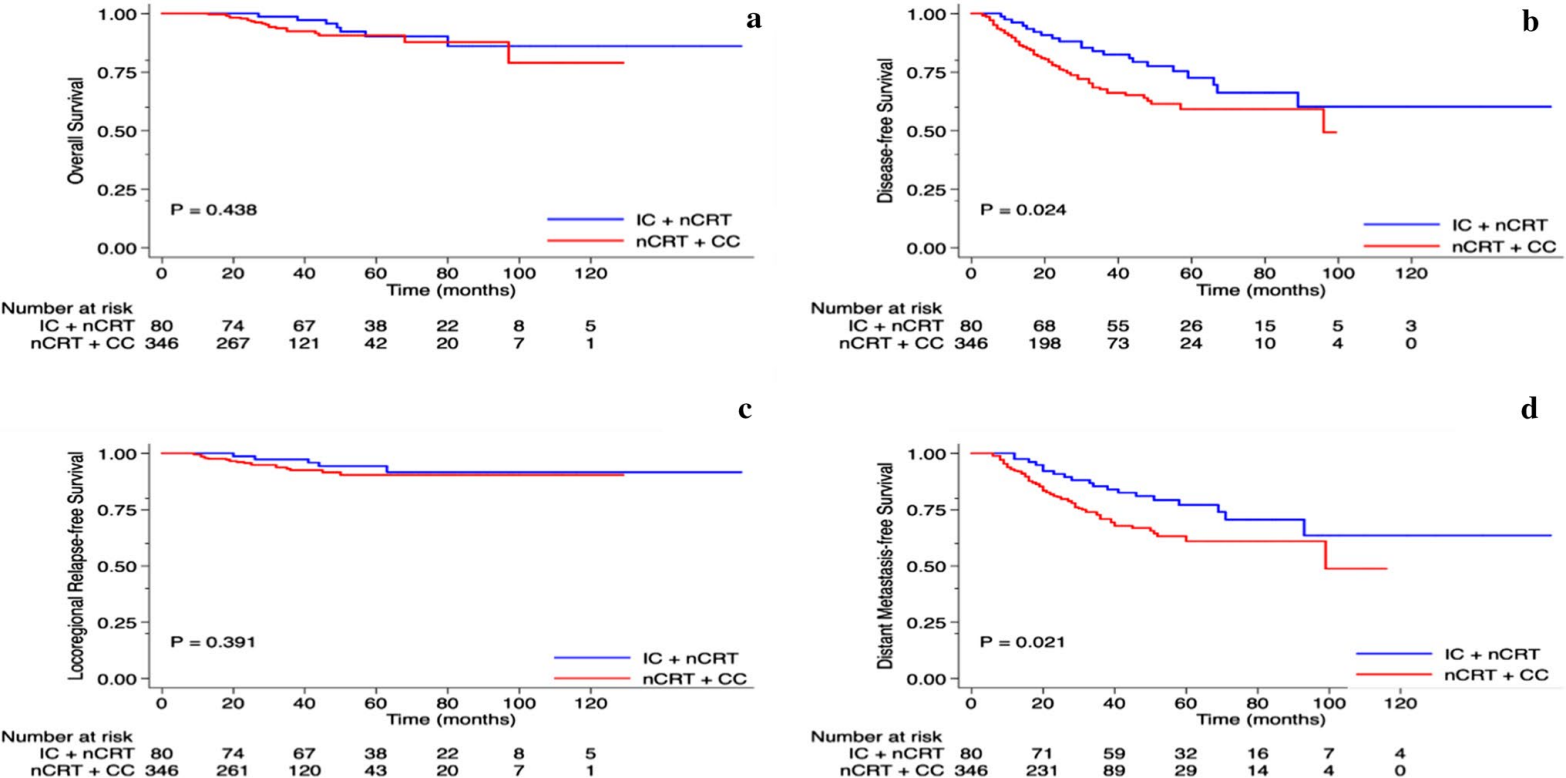
Kaplan–Meier analysis curves of 426 patients with newly diagnosed, biopsy-proven, locally advanced, non-metastatic rectal cancer (LARC), treated with neoadjuvant chemoradiotherapy (nCRT) and total mesorectal excision (TME), between January 2010 and December 2018, comparing those whose neoadjuvant chemotherapy (NCT) included induction chemotherapy (IC) and those whose NCT included consolidation chemotherapy (CC):** a** overall survival, **b** disease-free survival, **c** locoregional relapse-free survival, and **d** distant metastasis-free survival. Patients in the IC group had significantly better disease-free survival (*P* = 0.02) and distant metastasis-free survival (*P* = 0.02) than consolidation chemotherapy group patients. Differences between the 2 groups for overall survival rates and locoregional relapse-free survival rates were not significant

The median follow-up for all patients was 37 (range, 7 to 162) months. The 3-year OS, DFS, LRFS, and DMFS rates for all patients were 93.8%, 71.6%, 93.5%, and 74.4%, respectively. Patients in the IC group, relative to those in the CC group, demonstrated significantly better 3-year DFS (82.5% vs. 67.7%, *P* = 0.024) and DMFS (85.4% vs. 70.8%, *P* = 0.021) rates (Fig. 2). Patients in the IC group, relative to those in the CC group, also had higher 3-year OS (98.6% vs. 92.4%, respectively, *P* = 0.438) and LRFS (97.3% vs. 92.5%, respectively, *P* = 0.391) rates, but these differences were not significant.

**Fig. 3:**
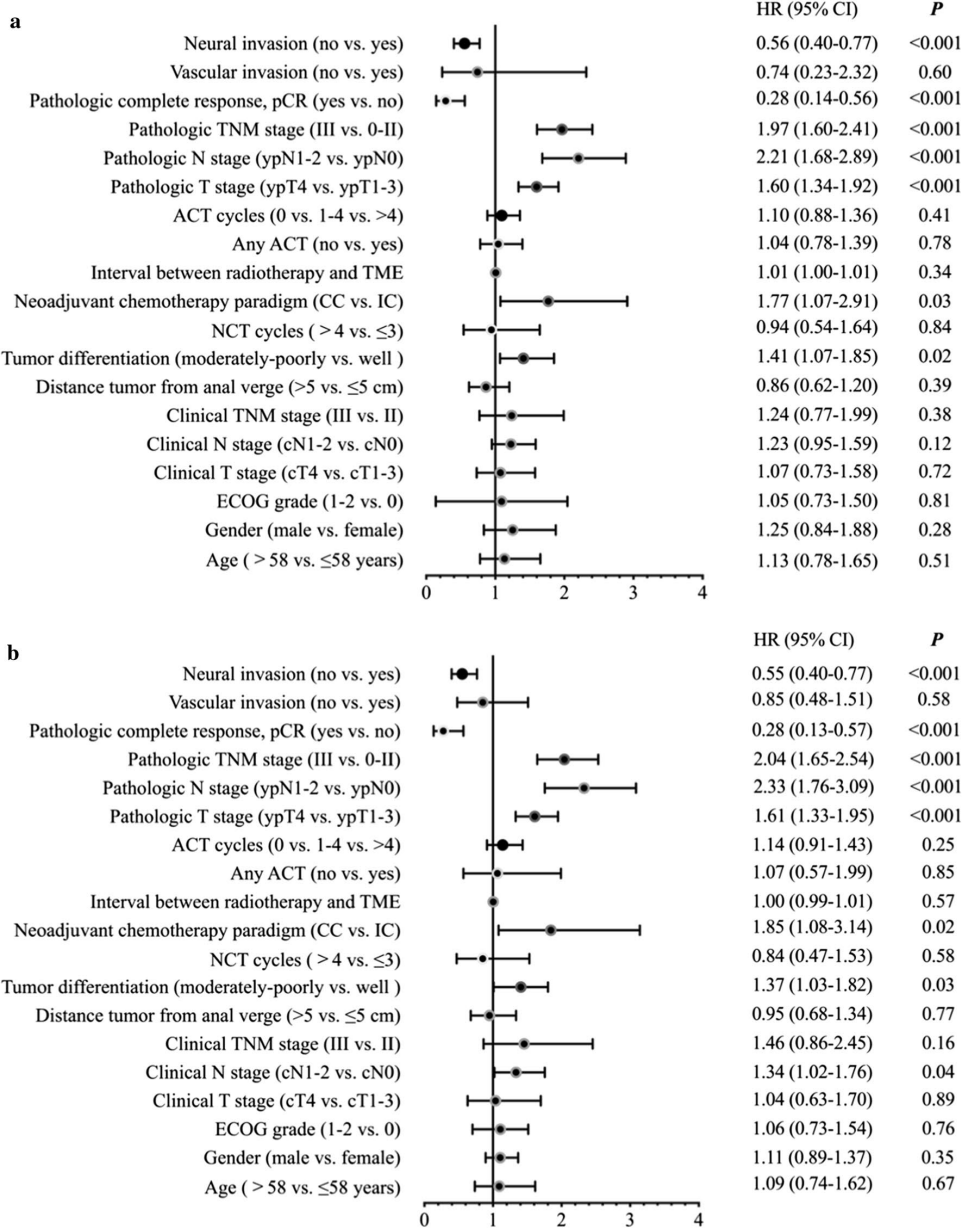
**a** Univariate analysis of risk of disease relapse for 426 patients with newly diagnosed, biopsy-proven, locally advanced, non-metastatic rectal cancer (LARC), treated with neoadjuvant chemoradiotherapy (nCRT) and total mesorectal excision (TME), between January 2010 and December 2018, by baseline, chemotherapy, and outcome characteristics. Results presented as hazard ratios (HR) with 95% confidence intervals (CI) for 3-year disease-free survival. Abbreviations: ACT, adjuvant chemotherapy; CC, consolidation chemotherapy; IC, induction chemotherapy; NCT, neoadjuvant chemotherapy; ECOG, European Cooperative Oncology Group. **b** Univariate analysis of risk of distant metastasis for 426 patients with newly diagnosed, biopsy-proven, locally advanced, non-metastatic rectal cancer (LARC), treated with neoadjuvant chemoradiotherapy (nCRT) and total mesorectal excision (TME), between January 2010 and December 2018, by baseline, chemotherapy, and outcome characteristics. Results presented as hazard ratios (HR) with 95% confidence intervals (CI) for 3-year distant metastasis-free survival). Abbreviations: ACT, adjuvant chemotherapy; CC, consolidation chemotherapy; IC, induction chemotherapy; NCT, neoadjuvant chemotherapy; ECOG, European Cooperative Oncology Group

Based on univariate analysis, the absence of neural invasion (HR = 0.56, *P* < 0.001) and pCR (HR = 0.28, *P* < 0.001) were significant predictors of DFS, whereas ypTN stage III vs. 0-II (HR = 1.97, *P* < 0.001), ypN1-2 vs. ypN0 stage (HR = 2.21, *P* < 0.001), ypT4 vs. ypT1-3 stage (HR = 1.60, *P* < 0.001), NCT paradigm of CC vs. IC (HR = 1.77, *P* = 0.03), and moderately-poorly vs. well differentiated tumor (HR = 1.41, *P* = 0.02) were significant predictors of disease relapse (Fig. 3a). Similarly, the absence of neural invasion (HR = 0.55, *P* < 0.001) and pCR (HR = 0.28, *P* < 0.001) were significant predictors of DMFS, whereas ypTN stage III vs. 0-II (HR = 2.04, *P* < 0.001), ypN1-2 vs. ypN0 stage (HR = 2.33, *P* < 0.001), ypT4 vs. ypT1-3 stage (HR = 1.61, *P* < 0.001), NCT paradigm of CC vs. IC (HR = 1.85, *P* = 0.02), moderately-poorly vs. well differentiated tumor (HR = 1.37, *P* = 0.03), and cN1-2 stage vs. cN0 (HR = 1.34, *P* = 0.04) were significant predictors of distant metastasis (Fig. 3b).

**Fig. 4:**
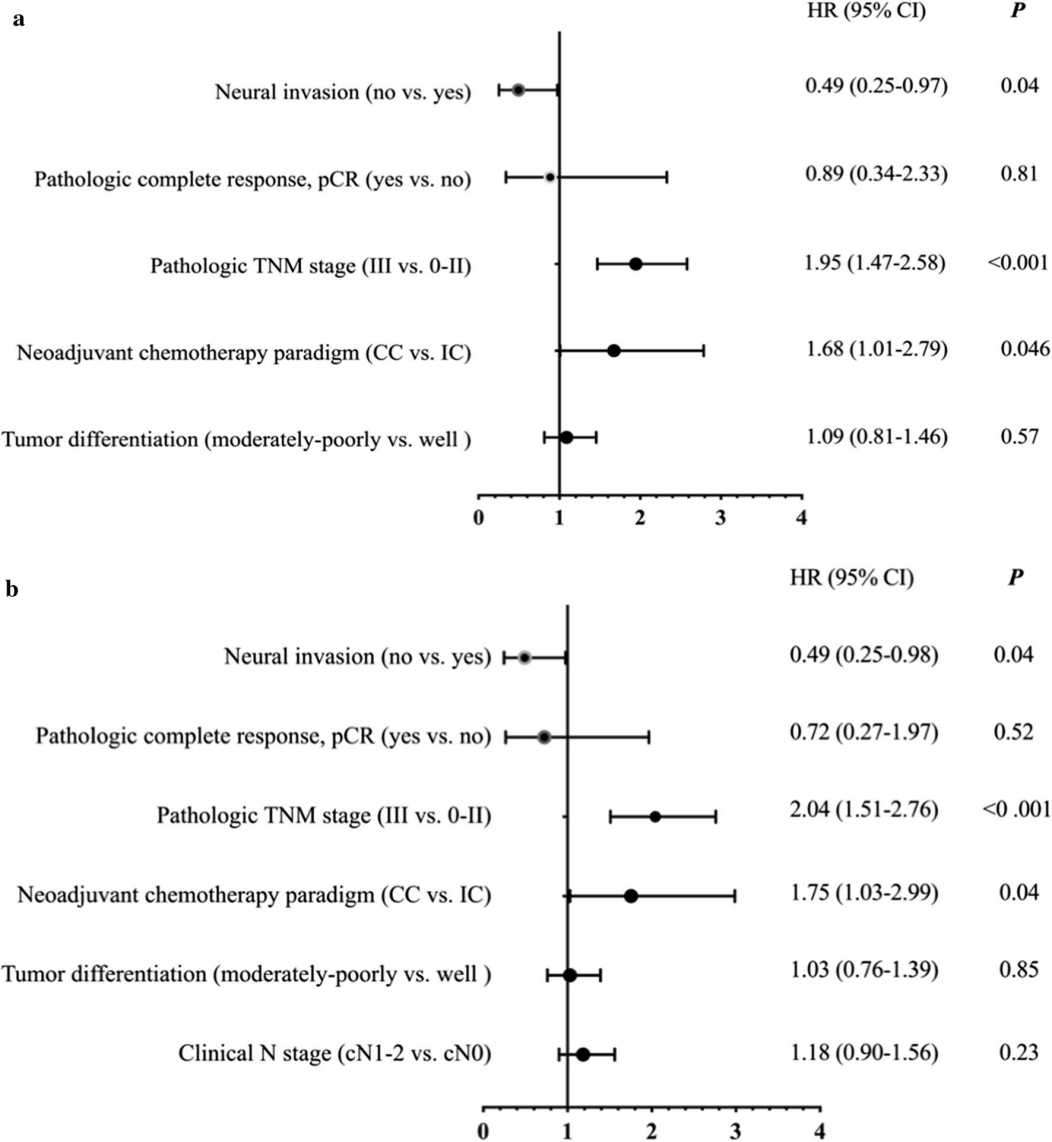
**a** Multivariate analysis of risk of disease relapse for 426 patients with newly diagnosed, biopsy-proven, locally advanced, non-metastatic rectal cancer (LARC), treated with neoadjuvant chemoradiotherapy (nCRT) and total mesorectal excision (TME), between January 2010 and December 2018, by the baseline, chemotherapy, and outcome characteristics that had significant results on univariate analysis. Results presented as hazard ratios (HR) with 95% confidence intervals (CI) for 3-year disease-free survival. Abbreviations: CC, consolidation chemotherapy; IC, induction chemotherapy. **b** Multivariate analysis of risk of distant metastasis for 426 patients with newly diagnosed, biopsy-proven, locally advanced, non-metastatic rectal cancer (LARC), treated with neoadjuvant chemoradiotherapy (nCRT) and total mesorectal excision (TME), between January 2010 and December 2018, by the baseline, chemotherapy, and outcome characteristics that had significant results on univariate analysis. Results presented as hazard ratios (HR) with 95% confidence intervals (CI) for 3-year metastasis-free survival. Abbreviations: CC, consolidation chemotherapy; IC, induction chemotherapy

Based on multivariate analysis, the absence of neural invasion was the only significant independent predictor of 3-year DFS (HR = 0.50, *P* = 0.04) (Fig. 4a) and of 3-year DMFS (HR = 0.49, *P* = 0.04) (Fig. 4b). Conversely, both ypTN stage III vs. 0-II and NCT paradigm of CC vs. IC were the only significant independent predictors of disease relapse (HR = 1.95, *P* < 0.001; HR = 1.68, *P* = 0.046; respectively) (Fig. 4a) and of distant metastasis (HR = 2.04, *P* < 0.001; HR = 1.75, *P* = 0.04; respectively) (Fig. 4b).

## Discussion

In this study, we compared the survival rates of patients with LARC who were treated with one of two different NCT paradigms, either induction chemotherapy prior to nCRT or nCRT followed by consolidation chemotherapy, before undergoing TME. We determined that those who received induction NCT had significantly better 3-year DFS and DMFS rates than those who received consolidation NCT. Whereas they also had better 3-year OS and LRFS rates, these differences were not statistically significant.

Recent studies focusing on the assessment of non-tissue sources of cancer, aim to identify the proper medical treatment before surgery is performed, thus allowing a tailored therapy based on cancer sequencing [19]. Moreover, the recent hospital centralization effect has reduced complications and post-operative morbidity for rectal cancer surgery possibly affecting oncological outcomes, too [20]. Over the decades, improved surgical techniques during TME and the addition of nCRT have dramatically reduced the risk of local recurrence for patients with LARC; however, distant metastases have remained a common area of failure for these patients [2–4]. Although ACT has been used to try to address this issue, patient compliance with ACT has historically been suboptimal and published clinical trials have not shown consistent benefit [5–7]. More recently, NCT has been employed in the preoperative setting in addition to the chemotherapy provided concurrently with radiotherapy during nCRT, in an effort to eliminate subclinical micrometastases and improve survival in patients with LARC. Relative to ACT, NCT has demonstrated significantly better patient compliance, ranging from 81.9% to 100%, and a superior toxicity profile [11, 21–24].

The FOWARC study is a multicenter, randomized, phase III study conducted at 15 hospitals in China [25]. The study aimed to evaluate superiority of the mFOLFOX-based treatment regimens versus fluorouracil-radiotherapy, in which patients with stage II/III rectal cancer were randomly assigned (1:1:1) to fluorouracil plus radiotherapy followed by surgery and ACT, the same treatment plus mFOLFOX6, or four to six cycles of mFOLFOX6 followed by surgery and ACT. The 3-year DFS was 72.9%, 77.2%, and 73.5% (*P* = 0.709), and the 3-year overall survival rate was 91.3%, 89.1%, and 90.7% (*P* = 0.971), respectively. In our study, patients in the IC group demonstrated slightly higher 3-year DFS (82.5%) and OS (98.6%) rates, compared with the fluorouracil plus radiotherapy followed by surgery and ACT group in their research. There is now a growing body of literature showing that the addition of NCT to nCRT and TME in patients with LARC may result in lower metastasis rates and better survival. The addition of NCT has been supported by a systematic review of 10 studies involving 648 patients with LARC who received induction NCT, nCRT, and surgical resection [24]. This review showed excellent results, including 5-year OS and DFS rates of 74.4% and 65.4%, respectively, as well as weighted mean local recurrence and distal failure rates of 3.5% and 20.6%, respectively. The results in our patients were consistent with these, with 3-year OS, DFS, LRFS, and DMFS rates for all patients of 93.8%, 71.6%, 93.5%, and 74.4%, respectively.

Some recent reports have begun to shed light on which NCT paradigm and how many cycles of NCT may be optimal. In the CAO/ARO/AIO-12 study, 306 patients with LARC were given either induction NCT before nCRT or consolidation NCT after nCRT [12]. They reported pCR in 17% of patients receiving induction and 25% of patients receiving consolidation NCT. However, the median interval between radiotherapy and TME was 45 days in their induction group and 90 days in their consolidation group [26]. We wonder if this difference in time intervals may have biased these pCR results, given that tumor regression after radiotherapy is time dependent. In our study, we found that the pCR rate for the patients receiving induction and consolidation did not differ significantly (22.5% vs. 18.5%, *P* = 0.41), and this was not biased by the median time interval between radiotherapy and TME, which was 56.5 days in the IC group and 56.0 days in the CC group. Also, even if pCR rates are better with consolidation than induction NCT, it is not clear whether this would translate into better metastasis-free or overall survival [13].

The OPRA trial involved patients with LARC who received nCRT and were randomized to 4 months of FOLFOX or CAPEOX before (Induction) or after (Consolidation) group [27]. Patients were re-staged 8 to 12 weeks after completing these treatments. Patients with complete or near-complete clinical response were offered a watch and wait strategy, while those with an incomplete response had TME. The proportion of patients who were offered watch and wait was significantly higher in the consolidation group than the induction group (58% vs. 43%, *P* = 0.01). The 3-year DFS and DMFS rates in the induction group (78% and 81%, respectively) and in the consolidation group (77% and 83%, respectively) were similar. These differ from the results in our study, in which the 3-year DFS and DMFS rates for patients who received induction NCT (82.5% and 85.4%, respectively) were significantly higher than those for patients who received consolidation NCT (67.7% and 70.8%, respectively).

One possible reason that our DFS and DMFS results differed from those in the OPRA trial is that a higher proportion of patients in our IC group than in our CC group received more than 4 cycles of NCT. In fact, this finding could be interpreted to suggest that patient compliance with NCT was higher in the IC group than the CC group in our study. This same concept about NCT compliance was used by the authors of the CAO/ARO/AIO-12 study, and they reported that compared to patients receiving consolidation NCT, a higher proportion of those receiving induction NCT received full doses of all recommended NCT cycles [12]. As the retrospective nature of our study, the actual compliance of these two paradigms is difficult to evaluate. We speculate that the inferior compliance with consolidation NCT in our study may have had an adverse effect on the DFS and DMFS rates of patients in the CC group. It is also possible that earlier exposure to systemic chemotherapy may have reduced the incidence of distant failure in the IC group. However, whether earlier administration of and good compliance with NCT translates directly into better long-term survival is still unknown.

This study has some limitations. First, aside from potential biases because it is a retrospective study from a single center, there were other potential sources of bias. For example, some imaging-relevant pretreatment characteristics we concerned were missed due to unavailable or poor-quality MRI scans, such as mesorectal fascia (MRF), extramural venous invasion (EMVI) and lateral lymph node (ILN). These prognostic factors can influence choices regarding NCT and may have led to selection bias. As another example, a significantly higher proportion of patients in the IC group, compared to those in the CC group, were younger and had clinical stage T4 rectal cancer. Although these differences could have biased our results, neither of these characteristics turned out to be significant prognostic factors in multivariate analysis. Second, there was considerable heterogeneity in the chemotherapy regimens (including ACT) and number of cycles of chemotherapy administered to patients in our study. This was in part because of the paucity of conclusive data concerning the use of NCT in LARC. Despite this, the regimens that we used for our patients were consistent with the National Comprehensive Cancer Network (NCCN) guidelines [1]. These differences could potentially have impacted the oncological outcomes in our study. However, it is noteworthy that the median total number of chemotherapy (combined NCT and ACT) cycles received was 8 in both of our study groups, and the heterogeneity of the treatment regimens was unavoidable given the retrospective nature of the study. Finally, although we did find better DFS and DMFS rates in the IC group, this cohort was relatively small. Because of this and the retrospective nature of our study, our findings should be interpreted with caution.

## Conclusion

Patients receiving nCRT and TME for LARC had better disease-free and metastasis-free survival with the addition of induction NCT rather than consolidation NCT to their treatment regimen.

## Data Availability

The datasets used for this current study are available from the corresponding author upon reasonable request.

## Abbreviations

LARC: Locally advanced rectal cancer
nCRT: Neoadjuvant chemoradiotherapy
TME: Total mesorectal excision
ACT: Adjuvant chemotherapy
DFS: Disease-free survival
OS: Overall survival
LRFS: Locoregional relapse-free survival
DMFS: Distant metastasis-free survival
pCR: Pathologic complete response
FOLFOX: Folinic acid, fluorouracil, and oxaliplatin
CAPOX: Capecitabine and oxaliplatin
Xeloda: Capecitabine
IMRT: Intensity-modulated radiotherapy
GTV: The gross tumor volume
CTV: The clinical target volume
AJCC: The American joint commission on cancer
NCCN: The national comprehensive cancer network
HR: Hazard ratios
CI: Confidence intervals
